# Intra-Articular Platelet-Rich Plasma Injections in Knee Osteoarthritis: A Review of Their Current Molecular Mechanisms of Action and Their Degree of Efficacy

**DOI:** 10.3390/ijms23031301

**Published:** 2022-01-24

**Authors:** Emérito Carlos Rodríguez-Merchán

**Affiliations:** 1 Department of Orthopedic Surgery, La Paz University Hospital, 28046 Madrid, Spain; ecrmerchan@hotmail.com; 2 Osteoarticular Surgery Research, Hospital La Paz Institute for Health Research—IdiPAZ (La Paz University Hospital—Autonomous University of Madrid), 28046 Madrid, Spain

**Keywords:** platelet-rich plasma, knee, osteoarthritis, mechanisms of action, efficacy

## Abstract

Knee osteoarthritis (OA) is estimated to affect more than 10% of the population, with a lifetime risk of 45%. Contemporary guidelines advise control of body weight, therapeutic physical exercise, drug treatment (oral non-steroidal anti-inflammatory drugs, paracetamol, opioids), and mechanical aids (walking aids, braces, orthoses). Nevertheless, these treatments typically have only short-term benefits. Intra-articular corticosteroids are typically advised, but only for short-term pain alleviation, given that their benefits last only a few weeks. The efficacy of hyaluronic acid is controversial. When the aforesaid options fail, total knee arthroplasty is generally recommended as an efficacious treatment. However, it is costly and can involve medical and postoperative complications. Therefore, determining alternate safe and effective treatments for knee OA is paramount. Platelet-rich plasma (PRP) has lately been investigated for the treatment of knee OA. This article reviews recent knowledge concerning PRP’s molecular mechanisms of action. The effectiveness of intra-articular PRP injections in the knee joint remains controversial, although most recent publications show pain alleviation in the short term. Orthopedic surgeons treating people with knee OA are becoming increasingly interested in PRP, despite indecisive clinical data and basic science information. Further studies comparing PRP with placebo are required.

## 1. Introduction

Primary knee osteoarthritis (OA) continues to be a hard-to-control degenerative disease. With the increase in average life expectancy and the prevalence of obesity, OA is creating a rising economic and physical burden [1]. Knee OA is a chronic musculoskeletal condition that can eventually require surgical intervention. Thus, patients continue to search for potential nonoperative therapies, such as platelet-rich plasma (PRP) injections into the affected knee [2,3].

According to Jayaram et al., PRP is an emergent therapeutic approach for the treatment of OA; however, there remains a lack of clinical evidence for its effectiveness, and its mechanisms of action are indeterminate [4]. Despite promising outcomes reported with regard to PRP utilization in knee OA, crucial issues such as conclusive evidence about its effectiveness, standard dose, and good preparation techniques remain unknown [5].

Knee OA is estimated to affect over 10% of the population worldwide [6], with a lifetime risk of 45% [7]. Contemporary guidelines advise both non-drug (such as exercise) and drug treatments, such as oral non-steroidal anti-inflammatory drugs (NSAIDs) [8,9]. Nevertheless, these treatments typically have only short-term benefits [10,11]. Moreover, the use of drugs is restricted in people with comorbidities due to the risk of complications [8].

Intra-articular corticosteroids are typically advised only for short-term pain alleviation because their benefits are limited to a few weeks [1,8,12], and repeated injections have been shown to be related to augmented cartilage loss [13]. Some authors have stated that the use of hyaluronic acid (HA) is controversial [8,9]. However, other authors have reported pain reduction after three to five weekly injections of HA lasting between 5 and 13 weeks (sometimes up to 1 year) [1].

When the aforementioned alternatives fail, total knee arthroplasty (TKA) is generally recommended as an effective treatment. However, it is costly and can involve medical and postoperative adverse effects [14]. Therefore, identifying alternative safe and effective therapies for knee OA is crucial.

Biological therapies have recently been investigated for the treatment of knee OA, such as PRP [15]. PRP is an autologous blood product with a high concentration of platelets. PRP’s effectiveness is thought to be related to the liberation of growth factors and other molecules, including platelet-derived growth factor (PDGF), transforming growth factor (TGF)-β, type I insulin-like growth factor (IGF-I), and vascular endothelial growth factor (VEGF) [16].

Some publications have stated that PRP might be promising for the treatment of knee OA [17,18,19]. Nevertheless, most disagree concerning the best methods and have many limitations that hamper an appropriate analysis of their outcomes, with risk of bias [18,20]. Heterogeneity in the preparation and injection methods employed by reported studies is a limitation for defining ideal PRP systems. Moreover, most trials have HA as a comparator, which is itself debatable [17]. Some trials have compared PRP with placebo, with outcomes showing significantly greater improvement in symptoms over saline at 6 and 12 months [21,22,23,24,25]. However, these trials had considerable methodological flaws, including lack of appropriate blinding, suggesting that the benefits might have been overvalued [17].

The advantages of PRP for the treatment of knee OA are the following: it is reasonably easy to use because its preparation is rapid and it is minimally invasive; it is a relatively affordable technique, thanks to use of existing public health service structures and equipment; and it is likely to be safe because it is an autologous product. Previous publications have reported only minor and transitory complications [17].

The purpose of this article is to review the current molecular mechanisms of action and the degree of efficacy of PRP intra-articular injections in patients with knee OA.

## 2. Platelet-Rich Plasma’s Molecular Mechanisms of Action

The results of a Cochrane Library and PubMed (MEDLINE) search of studies related to PRI in knee OA were analyzed. The searches were from the beginning of the search engines until 15 December 2021. Only the studies on PRP in knee OA that the author considered to be of most interest were included. PubMed found 454 articles, of which 80 were selected. One article was found in the Cochrane Library, which was also included, for a total of 80 references.

In 2011, a study was published stating that the use of growth factors (members of the TGF-β superfamily, fibroblast growth factor family, IGF-I, and PDGF) in the management of OA appeared promising [26].

In 2014, Sundman et al. reported that PRP treatment of OA joint tissues resulted in diminished catabolism; however, PRP caused a significant decrease in matrix metalloproteinase-13, an elevation in hyaluronan synthase-2 expression in synoviocytes, and an increase in cartilage synthetic activity. The findings of this study showed that PRP stimulates endogenous HA production and diminishes cartilage catabolism. PRP also suppressed inflammatory mediator concentration and expression of their genes in synovial and cartilage cells [27].

In 2015, a controlled laboratory study showed that PRP significantly stimulates cell proliferation and superficial zone protein secretion in cartilage and synovial cells of the human knee. These observations help explain the biochemical mechanisms related to the effectiveness of PRP in the treatment of knee OA [28].

In a murine OA model (controlled laboratory study) reported by Khatab et al. in 2018, multiple PRP releasate injections alleviated pain and synovial thickness, conceivably through the modulation of macrophage subtypes. Consequently, these injections appear to alleviate pain and synovial inflammation and might inhibit OA development in patients with early OA [29].

In 2018, a review of the literature on the PubMed database concluded that PRP therapy for OA appeared to exert modulation on the Wnt/β catenin pathway, which could be important in achieving its beneficial clinical effect [30].

In 2019, Liu et al., investigated the molecular mechanism of exosomes derived from PRP involved in alleviating OA. It is important to emphasize that exosomes play crucial roles in intercellular communication. In this study, primary rabbit chondrocytes were isolated and treated with interleukin (IL)-1β to establish the OA model in vitro. Proliferation, migration, and apoptosis assays were measured and compared between PRP-derived exosomes and activated PRP to assess the therapeutic effects on OA. The mechanism involving the Wnt/β-catenin signaling pathway was investigated by Western blot analysis. The therapeutic effects of PRP-derived exosomes on OA were found to be similar to or better than those of activated PRP in vitro and in vivo. Liu et al. stated that PRP-derived exosomes acting as carriers containing growth factors derived from PRP present a novel therapy for OA by activating the Wnt/β-catenin signaling pathway [31].

In a mouse model of post-traumatic OA reported in 2020, Jayaram et al. suggested that the effects of PRP on OA progression and disease-induced hyperalgesia might be leukocyte dependent. They also mentioned that leukocyte-poor PRP (LP-PRP) and to a lesser extent leukocyte rich-PRP (LR-PRP) protect against volume and surface loss [4].

The findings reported by Yang et al. in 2021 indicated that PRP abates IL-1β-induced chondrocyte apoptosis and inflammation at least partly through inhibiting hypoxia-inducible factor 2α [32].

In an in vivo OA model using PRP in rats, Sun et al. found that microRNA-337 and microRNA-375 were involved in delaying OA progression by affecting inflammation and apoptosis [33].

According to Sheean et al., the biologic activity of PRP is manifold: platelet α granules promote the release of various growth factors, including VEGF and TGF-β, and inflammation is modulated through the inhibition of the nuclear factor-κB pathway [34].

Uchiyama et al. studied the concentrations of humoral factors in PRP prepared from two kits and the impact of humoral factors on macrophage phenotypes. They found that the concentrations of cell components and humoral factors differed between PRP purified using the two kits. The autologous protein solution LR-PRP kit had a greater concentration of M1 and M2 macrophage-related factors. The addition of PRP supernatants to the culture media of monocyte-derived macrophages and M1 polarized macrophages showed that PRP suppressed M1 macrophage polarization and promoted M2 macrophage polarization [35].

In 2021, Szwedowski et al. described the growth factors liberated in the OA knee following PRP injection: tumor necrosis factor (TNF), IGF-1, TGF, VEGF, a disintegrin and metalloproteinase with thrombospondin motifs, interleukin, matrix metalloproteinase, epidermal growth factor, hepatocyte growth factor, fibroblast growth factor, keratinocyte growth factor, and platelet factor 4 [36].

**Figure 1:**
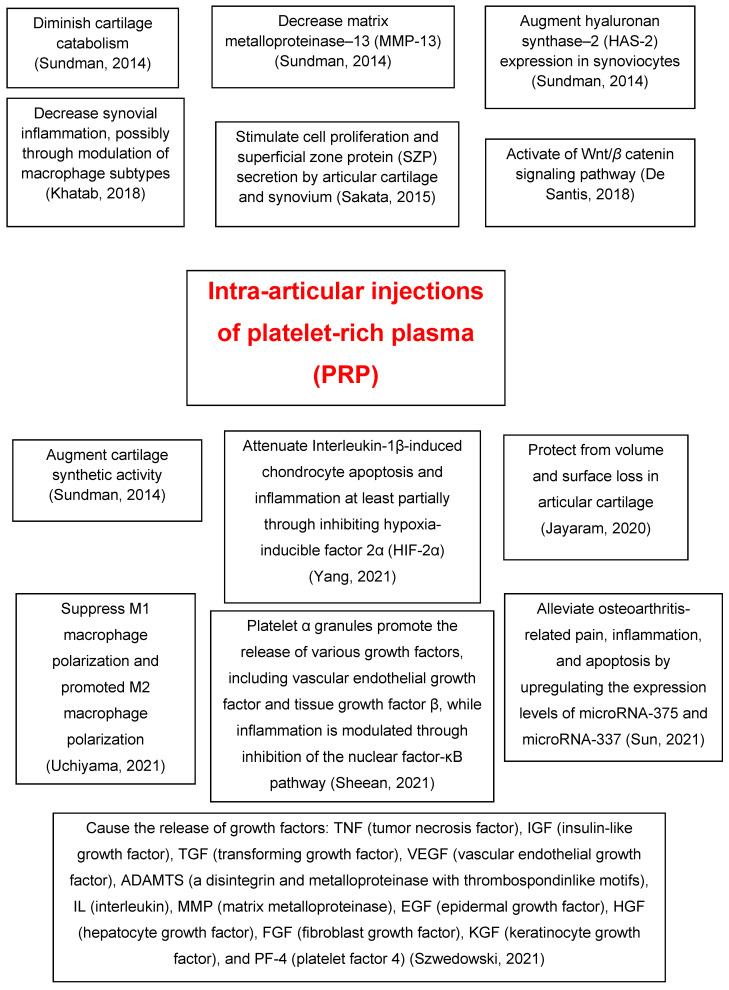
A summary of the molecular mechanisms of action of intra-articular injections of platelet-rich plasma (PRP) in knee osteoarthritis (OA) [4,27,28,29,30,32,33,34,35,36].

Table 1 [4,27,28,29,30,31,32,33,34,35,36] and Figure 1 summarize the molecular mechanisms of action of PRP intra-articular injections in knee OA.

## 3. Efficacy of Intra-Articular Platelet-Rich Plasma Injections in Knee Osteoarthritis

### 3.1. Placebo-Controlled Trials

In a single-blinded, randomized, placebo-controlled pilot study, Tucker et al. assessed molecular biomarkers and mesenchymal stem cells in synovial fluid during PRP treatment of the osteoarthritic knee joint [37]. Seventeen patients with mild-to-moderate knee OA were randomized in a 2:1 placebo-controlled ratio, receiving PRP or saline (placebo) intra-articular injection of the knee. Levels of IL-5, IL-6, IL-10, and TNF-α were measured in synovial fluid 10 days after PRP injection. Altered gene expression profiles in mesenchymal stem cells from patients receiving PRP were found for matrix metalloproteinases and inflammatory markers (IL-6, IL-8, CCL2, TNF-α). Alpha-2-macroglobulin protease was significantly augmented after PRP injection (*p* = 0.005). Western Ontario and McMaster Universities Arthritis Index (WOMAC) scores decreased for up to 3 months from baseline levels and remained low at 6 and 12 months in the PRP group. However, WOMAC scores for patients who received the saline injection were relatively unchanged for up to 12 months. Tucker et al. postulated that PRP modulates the local knee synovial environment by modifying the inflammatory milieu, matrix degradation, and angiogenic growth factors. The group receiving PRP had less pain and stiffness and improved function scores [37].

### 3.2. Randomized Controlled Trials

In a randomized controlled trial with level of evidence I, Yurtbay et al. observed that compared with placebo (sodium saline), LR-PRP injections were efficacious in the management of OA [38]. Multiple doses of PRP augmented the treatment efficacy and duration. The best results of PRP treatment were attained by patients aged 51–65 years, with a lower mechanical axis angle, and with Kellgren and Lawrence (K/L) grade 2 OA. The better group scores were found at 3 and 6 months. Patients who received PRP injections maintained better scores at 3, 6, and 12 months compared with the placebo group (*p* < 0.05). Multiple doses of PRP were found to be more efficacious than single-dose PRP at 6 and 12 months (*p* < 0.05). At 24 months, no significant score difference was found between groups. The most positive change in scores was found in those with K/L grade 2 OA, and the most positive change in range of motion (ROM) was found in those with K/L grade 3 OA. In the PRP groups, knee circumference diminished more at 1 and 6 months (*p* < 0.05) [38].

In a randomized, two-group, placebo-controlled, participant-, injector-, and assessor-blinded clinical trial reported in 2021, 288 individuals aged 50 years or older with symptomatic medial knee OA (K/L grade 2 or 3) were analyzed by Bennell et al. They used three intra-articular injections at weekly intervals of either LP-PRP (*n* = 140 participants) or saline placebo (*n* = 140 participants). Patients with symptomatic mild-to-moderate radiographic knee OA who had intra-articular injections of PRP were not found to have improved symptoms or joint structure at 12 months. Therefore, the authors did not support use of intra-articular injections of PRP for the treatment of knee OA [39].

Dório et al. evaluated the efficacy of intra-articular PRP and plasma to ameliorate pain and improve function in patients with knee OA over 24 weeks [40]. They performed a randomized, double-blind, placebo-controlled trial with three groups (*n* = 62): PRP (*n* = 20), plasma (*n* = 21), and saline (*n* = 21). Two ultrasound-guided knee injections were performed, with a 2-week interval. The primary parameter was visual analog scale (VAS) 0–10 cm for overall pain at week 24, with intermediate evaluations at weeks 6 and 12. The main secondary parameters were Knee Injury and Osteoarthritis Outcome Score (KOOS), Osteoarthritis Research Society International criteria, and timed up and go test (TUGT). At baseline, 92% of the participants were women, with a mean age of 65 years, mean body mass index (BMI) of 28 Kg/m^2^, and mean VAS pain of 6.2 cm. Changes in pain from baseline at week 24 were −2.9, −2.4, and −3.5 cm for PRP, plasma, and saline, respectively (*p* intergroup = 0.499). There were no differences among the three groups at weeks 6 and 12. Similarly, there were no differences among the groups regarding secondary outcomes. The PRP group showed a higher frequency of complications (65% versus 24% for plasma and 33% for saline, *p* = 0.02), mostly a mild transitory increase in pain. The conclusion of this study was that PRP and plasma were not better than placebo for pain reduction and function improvement in the knee with OA longer than 24 weeks. The PRP group had a greater frequency of mild transitory pain augmentation [40].

### 3.3. Systematic Reviews and Meta-Analyses

In 2021, Kim et al. performed a systematic review and meta-analysis (level of evidence IV) to assess the complications and clinical results of LP-PRP versus LR-PRP in knee OA. Intra-articular PRP injection resulted in improvements above the minimal clinically important difference in terms of pain and function in patients with knee OA up to 12 months. The risk of local complications appeared to be augmented following LR-PRP compared with LP-PRP injection. The findings of this study supported the use of intra-articular PRP injection for the management of knee OA [41].

A systematic review (level of evidence I) performed in 2021 by Nie et al. stated that PRP injections were beneficial for pain alleviation and functional improvement in patients with knee OA. However, they also stated that larger, randomized, high-quality studies were required to compare the effects of LP-PRP and LR-PRP [42].

Another review of the literature reported in 2021 by Li et al. included 959 patients with knee OA (1070 knees). The follow-up was between 3 and 12 months. PRP total knee scores were significantly better than baseline at 1, 2, 3, 6, and 12 months following PRP injection. Regarding complications, PRP did not increase the risk of adverse events compared with HA. Compared with many other treatment methods, the intra-articular injection of PRP was proven to be a safe and effective means of improving the quality of life of patients with knee OA [43].

Hong et al. compared the safety and effectiveness of PRP with placebo or other conservative treatments for knee OA (literature review and meta-analysis) [44]. Compared with placebo, PRP had a lower VAS score and a higher International Knee Documentation Committee (IKDC) subjective score at the sixth month following PRP injection and a significantly lower WOMAC score during the follow-up period. Compared with oral NSAIDs, PRP had a lower WOMAC score at the sixth month after injection. There were no significant differences in complications comparing PRP with placebo or HA. Different PRP applications did not show significant differences in VAS score in the first month or WOMAC score in the third month after treatment. There were no significant differences between triple PRP injection and single PRP injection in the short-term curative effect [44]. In a systematic review reported by Aiyer, they only recommended PRP for patients with early-stage OA (1 or 2) and who were aged younger than 65 years [45].

### 3.4. Case Series

In 2021, Moton et al. clinically evaluated the effectiveness of PRP for improving functional movement in knee OA (prospective case series) [46]. They analyzed 89 patients aged 30–65 years diagnosed with grade 1, 2, and 3 knee OA. PRP was administered in three doses 1 month apart, and patients were assessed for outcome measures after the third month of the third dose of PRP. To measure functional improvement in knee OA, the ROM, WOMAC, and VAS were utilized. PRP was injected into 89 patients, with a mean age of 61.24 years. The average preinjection WOMAC score was 37, and it declined to 18.8 following PRP (*p* < 0.02). The preinjection VAS was 8.42, which declined to 4.91, indicating mild-to-moderate pain. PRP treatment was appreciated by 63 (70.07%) patients, 17 (19.1%) patients were only partly satisfied, and 9 (10.1%) patients were dissatisfied. The outcomes of this study showed that the use of PRP injections for treating OA (grades 1 to 3) were shown to be successful in terms of improving functional results and diminishing pain intensity [46].

Sun et al. studied the effectiveness of a single intra-articular PRP injection for patients with early knee OA and considered subgroup analyses of radiographic severity and age [47]. Forty-one patients with knee OA (K/L grade 1–2) were given a single PRP injection into the knee and were evaluated at baseline and at 1, 3, and 6 months after injection. The primary parameter was the mean change from baseline in the VAS pain score (0–100 mm) at 6 months post-injection. Secondary parameters were the WOMAC, Lequesne index, single-leg stance test, use of rescue analgesics, and patients’ satisfaction. Thirty-eight patients completed the study. The mean pain VAS diminished significantly from 45.6 mm at baseline to 16.9, 14.0, and 15.5 mm at the 1, 3, and 6-month follow-ups, respectively (*p* < 0.001 for all). Significant improvements in WOMAC, Lequesne index, single-leg stance, and consumption of analgesics from baseline (*p* < 0.001 for all) were found at each follow-up. Patients’ satisfaction was high. No serious complications took place. Subgroup analyses showed that patients with grade 1 OA experienced a significantly greater VAS pain decrease at 3 months (*p* = 0.006) and 6 months (*p* = 0.005) than patients with grade 2 OA. The older age group (age > 60) showed significantly greater improvement in VAS pain, WOMAC function subscale scores, and total scores at 6 months postinjection compared with the younger age group (age ≤ 60). The younger age group reported better satisfaction at 1 and 3 months after injection. This study showed that PRP injection alleviated pain and improved function for 6 months for patients with early knee OA. Therefore, this study supported putting the one-injection regimen into clinical practice. However, Sun et al. stated that further research is required for more definitive conclusions [47].

Bec et al. retrospectively correlated PRP injections to the clinical responses of 75 patients with knee OA. Cartilage lesions were evaluated by utilizing magnetic resonance imaging (MRI) and the International Cartilage Regeneration Society classification. Patients’ subjective symptoms were recorded prior to injection and at 3 and 6 months following injection utilizing the KOOS. Responders were defined by a KOOS improvement of 10 points. At 6 months, 63.0% of the patients were responders. A single injection of PRP resulted in significant clinical improvement in the treatment of knee OA. Both baseline MRI and PRP biological features appeared to be factors predictive of the clinical response [48].

Hegaze et al. studied the impact of PRP on knee OA in a prospective cohort study involving 252 patients with various grades of knee OA. The K/L system was used for classifying the affected knee by degenerative cartilage lesions as well as early and severe OA. Pain was evaluated by the 0–10 Numeric Rating Scale, and knee ROM including flexion and extension was evaluated by goniometer. Follow-up evaluations were performed at 3-month intervals for a total of three visits. PRP injection was administered to all the patients, with a maximum of four injections. Intra-articular injections were found to provide significant pain relief and flexion improvement in patients with grades 2, 3, and 4 OA, especially with multiple injections in the short-term follow-up. Hegaze et al. recommended repeated injections, up to four times, because they appeared efficient in providing long-term alleviation of knee OA [49].

Rai et al. evaluated the safety and efficacy of intra-articular injection of autologous PRP in early knee OA. A total of 98 symptomatic individuals were given 2 injections of standardized PRP 3 weeks apart. Clinical results were assessed, utilizing the VAS and WOMAC questionnaire prior to treatment and at 6 weeks, 3 months, 6 months, and 1 year following treatment. Secondary objectives were safety (complications) and the impact of PRP on the various grades of knee degeneration. There was a statistically significant improvement in mean VAS and WOMAC scores at 6 weeks, 3 months, 6 months, and slight loss of improvement at the 1-year follow-up. A correlation between the degree of degeneration and the improvement in the mean scores was found. The reduction in mean pain score was greater in grades 1 and 2 (early OA) than in grade 3. The intra-articular injection of PRP was safe, with no major adverse events [50].

Jarayam et al. studied the effectiveness of LR-PRP as evaluated by functional and patient-reported outcomes at early time points (6 weeks). They analyzed 12 patients with diagnosed knee OA (K/L score of 2–3), who experienced a single ultrasound-guided LR-PRP injection. There was significant improvement in TUGT, pain and quality of life scales, and balance parameters. There were nonsignificant improvements in ROM and gait parameters. In patients with knee OA, LR-PRP demonstrated effectiveness in meaningful end points for functional and patient-reported outcomes at early time points [51]. Table 2 summarizes the efficacy of intra-articular PRP injections in knee OA [37,38,39,40,41,42,43,44,45,46,47,48,49,50,51].

## 4. Risk Factors Predictive of Failure of Platelet-Rich Plasma Injections

Alessio-Mazzola et al. examined the 5-year clinical effectiveness of PRP intra-articular injections in knee OA and investigated the risk factors predictive of treatment failure and poor clinical results [52]. They retrospectively assessed 118 patients treated with autologous PRP injection for low-to-moderate knee OA as demonstrated by X-ray and MRI, with a mean 51.1-month (range 29 to 89) follow-up. All the patients were assessed with Lysholm and WOMAC scores. By means of univariate and multivariate analyses, they analyzed the role of K/L grade, patellofemoral degeneration, age, BMI, relevant comorbidities, smoking status, sex, previous surgery, and conservative treatment. They found a significant improvement of all parameters at final follow-up and a high satisfaction percentage (79.7%). The overall failure percentage was 15.3% after a mean of 57.7 (range 33 to 85) months. The BMI and the K/L grade were found as significant independent risk factors related to failure. Patients younger than 60 had a significantly higher Tegner activity scale (*p* = 0.032) at final follow-up. Patients with K/L grade 3 and patients with patellofemoral MRI K/L grade 3 had significantly lower Lysholm scores (*p* = 0.026 and *p* = 0.042, respectively) at the final evaluation. Younger patients with lower BMI and lower radiographic OA grade had a significantly longer therapeutic benefit (*p* < 0.05). Intra-articular injections of PRP resulted in a significant clinical improvement in middle-aged adults with low-to-moderate-grade knee OA. BMI and high K/L grade were found as significant risk factors predictive of failure at mid-term follow-up [52].

PRP has been demonstrated to be effective in the setting of patellar tendinopathy and lateral epicondylitis. There is also considerable expectancy for the usefulness of PRP in managing partial hamstring tears and as an adjunct to rotator cuff repair, particularly in the setting of small-to-medium-sized tears, where it seems to exert ample analgesic effects and promote improved rates of rotator cuff repair healing [34].

Kikuchi et al. analyzed the short-term outcomes of LP-PRP and the factors that contribute to its effectiveness. They retrospectively reviewed 124 patients with knee OA. White blood cell (WBC) and platelet counts in the whole blood and the LP-PRP were measured. Knee OA severity was evaluated with radiography. A clinical evaluation was performed both before injection and after an average of 3.3 weeks following the injection, employing the Japanese Knee Osteoarthritis Measure. The responder rate was 58.1% and the contributing factors for responders were a greater VAS score prior to injection, WBC count in whole blood, and platelet concentration ratio of LP-PRP. The LP-PRP improved the clinical scores in the short term [53].

## 5. Single- vs. Multi-Dose Intra-Articular Injection

A study compared the efficacy of single, double, and triple doses of intra-articular PRP in early stages of knee OA. This single-blind, randomized, superiority trial included 180 knees of 90 patients (22 males, 68 females; mean age 47.9 years) with bilateral OA knee of K/L grade 1–2. The patients were randomized (30 in each group) to receive single, double, or triple doses of intra-articular PRP (2 weeks apart in repeat injections). The outcome measures were VAS, IKDC, KOOS, and Tegner Lysholm Knee Score. The scores were collected prior to intervention and at 6 weeks, 3 months, 6 months, and 1 year after the intervention. All three groups were comparable with respect to demographic and disease characteristics. All four scores were comparable among the three groups prior to intervention and at 6 weeks, 3 months, and 6 months. However, at 1-year follow-up, the three-dose group showed superiority to others in terms of all four scores. All three groups showed improvements until 6 months and deterioration thereafter, which was only marginal in the three-dose group. All groups showed a statistically significant improvement of scores compared with baseline scores at 1 year. There were no major adverse events. The intra-articular administration of three doses of PRP yielded results superior to single and double doses at the end of 1 year. It was stated that repeat doses are probably required to sustain the benefit accomplished at 1 year [54].

## 6. Two vs. Four Intra-Articular Injections

Ngarmukos et al. compared two and four intra-articular injections of PRP in terms of changes in synovial cytokines and clinical results [55]. They analyzed 125 patients with knee OA who were administered PRP injections at a 6-week interval. Prior to each PRP injection, synovial fluid aspiration was collected for investigation. Patients were divided into two groups receiving either two or four intra-articular PRP injections (groups A and B, respectively). Changes in synovial biomarkers were compared with the baseline levels of both groups, and clinical results were assessed until 1 year. Ninety-four patients who had completed synovial fluid collection were included for final assessment, 51 in group A and 43 in group B. There were no differences in mean age, sex, BMI, or radiographic OA grading. The average platelet count and WBC count in PRP were 430,000 and 200/µL, respectively. There were no changes in synovial inflammatory cytokines (IL-1β, IL-6, IA-17A, and TNF-α), anti-inflammatory cytokines (IL-4, IL-10, IL-13, and IL-1RA), or growth factors (TGF-β1, VEGF, PDGF-AA, and PDGF-BB) between baseline levels and 6 weeks in group A, and 18 weeks in group B. Both groups had significantly improved clinical results from 6 weeks, including VAS and patient-reported outcome measures (WOMAC and Short Form-12), with a significant delayed improvement of performance-based measures (TUGT, five-time sit-to-stand test, and 3-min walk test). Ngarmukos et al. stated that neither two nor four PRP intra-articular injections at a 6-week interval for knee OA showed changes in synovial cytokines and growth factors, but they similarly improved clinical results from 6 weeks until 1 year [55].

## 7. T2-Mapping Magnetic Resonance Imaging Evaluation of Cartilage Is a Valuable Indicator for Treatment-Related Changes over Time

A study evaluated the ability of T2 mapping using 3T MRI in addition to morphological sequences to assess the effectiveness of PRP injections, characterizing qualitatively and quantitatively the grade of knee cartilage repair in patients with patellofemoral chondropathy [56]. Cobianchi Bellisari et al. retrospectively studied 34 patients (22 men, 12 women, mean age 41.8 years, including 22 men), who experienced intra-articular PRP injections and completed a clinical and instrumental follow-up. As a control group, they assessed 34 patients who experienced nonsurgical treatment. All patients were analyzed by means of VAS, WOMAC, and imaging studies with 3T MRI cartilage analysis with T2 mapping sequences for cartilage analysis prior to and following injection. In the study group, mean pretreatment T2 relaxation time values were 44.2 ms, considering all articular cartilage compartments, with a significant decrease at follow-up (*p* < 0.001). For the index compartment, mean preinjection T2 relaxation time was 47.8 ms, with a significant decrease at follow-up (*p* < 0.001). Assessment of focal cartilage lesions reported a preinjection mean T2 value of 70.1 ms and a postinjection mean value of 59.9 ms (*p* < 0.001). Preinjection WOMAC and VAS scores were 18.3 and 7, respectively; the postinjection values were 7.3 and 2, respectively (*p* < 0.001). In the control group, despite clinical improvement, no significant T2 value change during the follow-up period was found. It was stated that T2 mapping is a valuable indicator for chondropathy and treatment-related changes over time [56].

## 8. Other Important Considerations

### 8.1. The Effect of Platelet-Rich Plasma on Cartilage Thickness

In 2021, Baki et al. analyzed the impact of PRP intra-articular injections on cartilage thickness in individuals with primary knee OA. A total of 100 patients with mild-to-severe primary knee OA using the K/L grading scale were included and divided into two groups. Group I included 50 patients who experienced two intra-articular knee injections of PRP, 1 week apart; group II included 50 patients who were given NSAIDs and chondroprotective drugs. Functional evaluation of patients was performed employing the basal WOMAC score at 2 and 6 months. Ultrasonography evaluation of femoral condylar cartilage thickness was performed basally and at 6 months. Greater improvement of WOMAC score was found at 2 and 6 months in group I after PRP injection compared with group II (*p* values < 0.001). The improvement of WOMAC in group I occurred for all severity degrees of OA (*p* < 0.001). A significant increase in cartilage thickness was encountered at the intercondylar area at 6 months relative to baseline evaluation by ultrasonography in group I (*p* = 0.041). Treatment with PRP injections relieved pain and improved knee function in patients with various degrees of knee OA [57].

### 8.2. The Correct Dose of Platelet-Rich Plasma Is Crucial for Long-Term Clinical Effectiveness

In 2021, Bansal et al. demonstrated that an absolute count of 10 billion platelets is critical in a PRP formulation to have a long sustained chondroprotective effect up to 1 year in moderate knee OA [5].

### 8.3. The Effect of Leukocyte Concentration

Abbas et al. performed a meta-analysis of the literature comparing LP-PRP or LR-PRP in a study with level of evidence 2. The parameters evaluated were the change in the WOMAC score between baseline and follow-up, changes in the WOMAC pain subscale, VAS for pain, IKDC subjective score between baseline and follow-up, and the frequency of local complications. This meta-analysis included 2260 patients, with an average follow-up period of 9.9 months. No significant (*p* < 0.05) difference in any parameters or local complications between LP-PRP and LR-PRP was encountered. The surface under the cumulative ranking showed that, for all parameters, LP-PRP was preferred to LR-PRP across follow-up periods. However, leukocyte concentrations of PRP did not play a significant role in patient-reported outcome measures for knee OA [58].

### 8.4. The Immature Platelet Fraction Affects the Efficacy

According to Uchino et al., PRP effectiveness for OA might depend on the patient’s biological status. They analyzed blood cell counts, including the immature platelet fraction (IPF), in the peripheral blood and PRP of 144 individuals with knee OA who received PRP treatment. The mean leukocyte and platelet concentrations in whole blood and PRP were determined with an XN-1000 automated hematology analyzer. VAS scores and KOOS prior to and 1 month following a single PRP injection were also recorded. Higher platelet and lower leukocyte concentration rates were found in PRP compared with whole blood. The platelet concentration in whole blood was negatively correlated with VAS amelioration. The percentage of IPF (IPF%) in whole blood was positively correlated with VAS amelioration and KOOS (pain) improvement, whereas the IPF% in PRP tended to correlate only with VAS amelioration. Moreover, multivariate logistic regression showed that a high IPF% in whole blood was significantly associated with VAS amelioration. A low percentage of neutrophils in PRP was significantly associated with VAS amelioration and KOOS amelioration [59].

### 8.5. Platelet-Rich Plasma Injections Delay the Need for Total Knee Arthroplasty

Sánchez et al. studied whether PRP could postpone or even avoid TKA in patients with knee OA. They analyzed the length of delay and the percentage of patients without TKA. The study included 1084 patients, 667 of whom met the inclusion criteria. Some 74.1% of the patients in the retrospective study achieved a delay in the TKA of more than 1.5 years, with a median delay of 5.3 years. The survival analysis showed that 85.7% of the patients did not undergo a TKA during the 5-year follow-up. The severity degree, age, PRP cycles, and administration route had a statistically significant impact on the effectiveness of PRP in delaying surgery. The findings of this study suggested that PRP intra-articular injections in knee OA can delay TKA [60].

## 9. Discussion

PRP is an autologous concentrate of platelets and growth factors, derived from centrifugated blood [61,62,63]. There are two other types of platelet concentrates: platelet-rich fibrin and plasma-rich growth factors [64]. PRP can be attained only from liquid blood; it is impossible to attain PRP from serum or clotted blood [65].

There are different commercial techniques to collect blood and to attain PRP. The differences among them include the amount of blood needed to be taken from the patients; the isolation technique; the speed of centrifugation; the amount of attained concentrated volume after centrifugation; processing time; increase in platelets; and platelet capture effectiveness [66].

It has also been reported that different techniques of blood centrifugation affect the leukocyte ratios [67]. The number of platelets in 1 μL of blood in healthy individuals ranges from 150,000 to 300,000 [68]. Platelets are responsible for hemostasis [61,68].

Alpha granules of platelets contain different types of proteins, such as growth factors (e.g., transforming growth factor β, insulin-like growth factor, epidermal growth factor), chemokines, coagulants, anticoagulants, fibrinolytic proteins, adhesion proteins, integral membrane proteins, immune mediators, angiogenic factors and inhibitors, and microbicidal proteins [65,68,69].

The exact mechanism of PRP action remains unknown [70,71]. PRP appears to stimulate the chondrocytes to engineer the cartilage and the biosynthesis of collagen and proteoglycans [72]. It has been used in various medical specialties, such as oral and maxillofacial surgery (including temporo-mandibular joint OA), dermatology, ophthalmology, cardiothoracic surgery, and plastic surgery [73,74].

Several randomized controlled trials have shown the superiority of PRP over both corticosteroids and HA in treating knee OA-related symptoms [34]. In 2021, Cohen et al. utilized the Google Trends tool to provide a quantitative analysis of national interest in PRP injections for knee OA. They demonstrated a significant increase in Google queries related to PRP injections for OA of the knee since 2009 [2]. According to Andia et al., intra-articular injections of PRP can provide pain alleviation and potential benefits in OA progression. Meta-analyses have shown the clinical superiority of PRP compared with other intra-articular injections; however, the results were modest and the effect sizes were small [75].

Zhair et al. have developed an integrative workflow to assess responses to PRP in vitro, and to evaluate whether the in vitro responses to PRP were associated with the PRP composition and the clinical results in patients with knee OA. Based on the patient-reported results and accomplishment of minimal clinically important differences in patients with OA undergoing PRP injections, they identified responders and nonresponders to the treatment. Comparisons of PRP from these patient groups allowed them to identify differences in the composition and in vitro activity of PRP [76].

In a consensus statement, the main recommendations of French-speaking experts concerning the utilization of intra-articular PRP injections in knee OA were the following: (1) PRP was effective for the treatment of early to moderate knee OA; (2) A PRP treatment sequence in knee OA can include one–three injections; (3) LP-PRP should be preferred in knee OA; (4) PRP should not be mixed with an anesthetic or intra-articular corticosteroids. However, many of the aforementioned recommendations had a very low level of evidence and were mainly based on the clinical experience of the experts [77].

Chan et al. stated that randomized controlled trials for PRP injections in knee OA were not robust in quality [78]. According to Baki et al., intra-articular PRP injections can alleviate pain and improve knee function in patients with various grades of OA [57]. In comparison with placebo, Yurtbay et al. found that LR-PRP injections were efficacious in the management of OA. Multiple doses of PRP augmented the treatment effectiveness and duration. The best outcomes were attained by individuals aged 51–65 years, with lower mechanical axis angle, and by patients with K/L stage 2 OA [38]. However, the study by Bennell et al. showed that among patients with symptomatic mild-to-moderate radiographic knee OA, intra-articular injection of PRP did not result in a significant difference in symptoms at 12 months compared with saline injection (placebo) [39].

According to Szwedowski et al., various experimental and clinical studies have demonstrated the positive effect of PRP on structural modulation and its anti-inflammatory effects on knee OA [36]. In a recent meta-analysis of randomized controlled trials (level of evidence I), PRP demonstrated the best overall result compared with corticosteroids, HA, and placebo for individuals with knee OA at 3, 6, and 12 months’ follow-up. However, no discrepancies were detected among corticosteroids, HA, and placebo [79].

In 2019, Whittle et al. published a protocol for a Cochrane Review with the following objectives: to evaluate the benefits and costs of autologous PRP for people with knee OA, in terms of pain, function, management success, quality of life, disease progression, and complications [80].

## 10. Conclusions

The effectiveness of intra-articular PRP injections for knee joint OA is still controversial, although most recent publications show pain relief in the short term. In a recent meta-analysis of randomized controlled trials (level of evidence I), PRP demonstrated the best overall result compared with corticosteroids, HA, and placebo for individuals with knee OA at 3, 6, and 12 months. Orthopedic surgeons treating people with knee OA are becoming increasingly interested in PRP, despite indecisive clinical data and basic science information. Further studies comparing PRP with placebo are required.

## Figures and Tables

**Table 1:** Summary of PRP’s molecular mechanisms of action.

Authors	Year	Main Findings
Sundman et al. [27]	2014	PRP treatment decreases catabolism and matrix metalloproteinase-13 and increases hyaluronan synthase-2 expression in synoviocytes and cartilage synthetic activity.
Sakata et al. [28]	2015	PRP stimulates cell proliferation and superficial zone protein secretion by articular cartilage and synovium of the human knee joint.
Khatab et al. [29]	2018	Multiple PRP releasate injections reduce pain and synovial thickness, possibly through modulation of macrophage subtypes.
De Santis et al. [30]	2018	PRP therapy for OA exerts modulation on the Wnt/*β* catenin pathway that might be relevant in achieving its beneficial clinical effect.
Liu et al. [31]	2019	The therapeutic effects of exosomes derived from PRP on OA were similar or better compared with those of activated PRP in vitro or in vivo.
Jayaram et al. [4]	2020	The effects of PRP therapy on OA progression and disease-induced hyperalgesia might be leukocyte-dependent.
Yang et al. [32]	2021	PRP attenuates interleukin-1β, inducing chondrocyte apoptosis and inflammation at least partially through inhibiting hypoxia-inducible factor 2α.
Sun et al. [33]	2021	Micro-RNA (miR)-337 and miR-375 are involved in PRP-delayed OA progression by affecting inflammation and apoptosis.
Sheean et al. [34]	2021	Platelet α granules promote the release of various growth factors, including vascular endothelial growth factor and tissue growth factor β, and inflammation is modulated through inhibition of the nuclear factor-κB pathway.
Uchiyama et al. [35]	2021	The autologous protein solution leukocyte-rich PRP kit has a higher concentration of M1 and M2 macrophage-related factors.
Szwedowski et al. [36]	2021	Growth factors released in the OA knee joint after PRP injection: tumor necrosis factor, insulin-like growth factor, transforming growth factor, vascular endothelial growth factor, a disintegrin and metalloproteinase with thrombospondin motifs, interleukin, matrix metalloproteinase, epidermal growth factor, hepatocyte growth factor, fibroblast growth factor, keratinocyte growth factor, and platelet factor 4.

PRP = platelet-rich plasma; OA = osteoarthritis.

**Table 2:** Summary of the efficacy of intra-articular PRP injections in knee OA.

Authors	Year	Type of Study	Main Findings
Tucker et al. [37]	2021	Single-blinded, randomized, placebo-controlled pilot study	The PRP treatment group had less pain and stiffness and improved function scores than the placebo (saline) group
Yurtbay et al. [38]	2021	Randomized, double-blind, placebo-controlled clinical trial	Compared with placebo (sodium saline), LR-PRP treatment was effective in the treatment of OA. Multiple doses of PRP increased the treatment efficacy and duration. Patients aged 51–65 years scored better at 6 months
Bennell et al. [39]	2021	Randomized, 2-group, placebo-controlled, participant-, injector-, and assessor-blinded clinical trial	Among patients with symptomatic mild-to-moderate radiographic knee OA, intra-articular PRP injection, compared with injection of saline placebo, did not result in a significant difference in symptoms or joint structure at 12 months.
Dório et al. [40]	2021	Randomized, double-blind, placebo-controlled trial of 3 groups of patients: PRP, plasma, and saline.	There were no differences among the 3 study groups at weeks 6 and 12.
Kim et al. [41]	2021	Systematic review and meta-analysis (level of evidence IV)	Intra-articular PRP injection resulted in improvements above the minimal clinically important difference in terms of pain and function up to 12 months.
Nie et al. [42]	2021	Meta-analysis of randomized controlled clinical trials (level of evidence I)	PRP injections were beneficial for pain alleviation and functional improvement in knee OA.
Li et al. [43]	2021	Literature review	Compared with many other treatment methods, intra-articular injection of PRP proved to be safe and effective to improve the quality of life of patients with knee OA.
Hong et al. [44]	2021	Systematic review and meta-analysis	Compared with placebo, PRP had a lower VAS score and higher IKDC subjective score at 6 months after treatment and a significantly lower WOMAC score during the follow-up period.
Aiyer et al. [45]	2021	Systematic review of clinical studies	These authors recommended PRP for patients with early-stage OA (I or II) and who are aged younger than 65.
Moton et al. [46]	2021	Prospective case series	PRP injections for treating OA (grade 1 to 3) proved to be successful in terms of improving functional outcomes and reducing pain intensity.
Sun et al. [47]	2021	Case series	One injection of PRP improved pain and function for 6 months for patients with early knee OA.
Bec et al. [48]	2021	Case series (retrospective study)	A single injection of pure PRP resulted in significant clinical improvement in the management of knee OA.
Hegaze et al. [49]	2021	Prospective case series	Intra-articular injections gave significant pain and flexion improvement in patients with grades II, III, and IV OA, especially with multiple injections in the short-term follow-up.
Rai et al. [50]	2021	Case series	PRP was a safe and effective therapy for early OA knees. It provided a significant clinical improvement in patients, with some loss of improvement with time.
Jayaram et al. [51]	2021	Case series	LR-PRP demonstrated efficacy in meaningful end points for functional and patient-reported outcomes at early time points in patients with knee OA.

PRP = platelet-rich plasma; LR-PRP = leukocyte rich PRP; OA = osteoarthritis; VAS = visual analog scale; IKDC = International Knee Documentation Committee; WOMAC = Western Ontario and McMaster Universities Osteoarthritis Index.

## Data Availability

Not applicable.
